# Development of rat metabolic syndrome models: A review

**DOI:** 10.14202/vetworld.2021.1774-1783

**Published:** 2021-07-07

**Authors:** Shirly Gunawan, Ahmad Aulia, Vivian Soetikno

**Affiliations:** 1 Department of Pharmacology, Faculty of Medicine, Universitas Tarumanagara, Jakarta, Indonesia; 2 Doctoral Programme in Biomedical Science Faculty of Medicine, Universitas Indonesia, Jakarta, Indonesia; 3 Department of Histology, Faculty of Medicine, Universitas Indonesia, Jakarta, Indonesia; 4 Department of Pharmacology and Therapeutics, Faculty of Medicine, Universitas Indonesia, Jakarta, Indonesia

**Keywords:** high-fat diet, high-fructose diet, high-sucrose diet, metabolic syndrome, rat models

## Abstract

Metabolic syndrome (MetS) has become a global problem. With the increasing prevalence of MetS worldwide, understanding its pathogenesis and treatment modalities are essential. Animal models should allow an appropriate representation of the clinical manifestations of human conditions. Rats are the most commonly used experimental animals for the study. The development of a proper MetS model using rats will contribute to the successful application of research findings to the clinical setting. Various intervention methods are used to induce MetS through diet induction with various compositions, chemicals, or a combination of both. This review will provide a comprehensive overview of several studies on the development of rat MetS models, along with the characteristics of the clinical manifestations resulting from each study.

## Introduction

Metabolic syndrome (MetS), also known as syndrome X, is a collection of metabolic disorders, including hypertension, glucose metabolism disorders, insulin resistance, obesity, and dyslipidemia [1]. Each MetS component is an independent risk factor for cardiovascular disease, and the combination of these risk factors elevates the severity of cardiovascular disease [2]. Furthermore, these components and their combination increase the risk of type 2 diabetes mellitus [3]. The American Heart Association/National Heart, Lung, and Blood Institute and the National Cholesterol Education Program Adult Treatment Panel III have established that MetS is diagnosed when three or more of the following criteria are present: Waist circumference of more than 102 cm in men and more than 88 cm in women, fasting plasma glucose of more than or equal to 100 mg/dL (5.6 mmol/L), systolic blood pressure of more than or equal to 130 and diastolic blood pressure of more than or equal to 85 mmHg, triglyceride plasma levels of more than or equal to 150 mg/dL (1.7 mmol/L), and high-density lipoprotein (HDL) cholesterol levels of <40 mg/dL (1.03 mmol/L) in men and <50 mg/dL (1.29 mmol/L) in women [1]. MetS has become a global health problem. Worldwide, the prevalence of MetS reaches 20%–30% and increases with age in a sex-specific manner [4].

With the increasing prevalence of MetS, understanding its pathogenesis and treatment modalities using animal models are essential. The development of proper models has contributed to the successful application of research findings to the clinical setting. Animal models should allow an appropriate representation of the clinical manifestations of human conditions. Rats (*Rattus norvegicus*) have been used as animal models for investigating MetS [5-7]. However, no experimental rat model has become the standard reference for MetS models. Studies have used various intervention methods to induce MetS, whether through diet induction with various compositions, chemicals, or a combination of both.

This review provides the readers a comprehensive overview of several studies on developing rat MetS models, along with the characteristics of clinical manifestations resulting from each study. Some factors could influence the study results, including the type of diet, duration of induction, strain, and gender and age of the animals at the beginning of induction.

## Rats as Animal Models

To date, rats, mice, dogs, pigs, and rabbits have been used as animal models [5]. However, not all studies can produce clinical manifestations of MetS as in humans. Rats and mice from the rodent group are the most frequently used models to replicate human disease’s phenotype and pathogenesis, especially hypertension, diabetes, and obesity [6]. The rodent group could tolerate well to various types of diets. Wistar and Sprague-Dawley outbred rats can be considered the most common rodents used to develop MetS models since they are susceptible to diet-induced obesity and resistance with individual characteristics [8]. Besides the ease of handling, their larger size than mice simplifies measuring some metabolic parameters, such as blood pressure [5,8]. Nevertheless, they occasionally present some drawbacks because some strains do not develop all MetS components.

Many variables should be considered when using rats as animal models [9]. In addition to strain, the age of the animals contributes to the successful development of MetS models, as metabolism changes substantially from pre-adult age to adult age. Cheng *et al*. [10] have shown that rats fed with a high-fat diet (60% kcal) produced a complete phenotype of MetS, namely, increased body weight, fat mass, fasting plasma glucose, hypertriglyceridemia, hepatic steatosis, and hypertension. The rats are induced at weaning age (3 weeks). All manifestations occurred more rapidly (4 weeks vs. 6 weeks) than induction performed in adult rats (8 weeks of age). Induction duration is also a strong determinant of the metabolic outcome [9]. Sexual dimorphism due to diet-induced insulin resistance and glucose intolerance is also observed in rats, with males being the most affected [9].

Studies using a MetS model can also be conducted using genetically modified animals, for example, leptin-deficient mice, leptin receptor-deficient mice, Zucker fatty rats, Zucker diabetic fatty rats, DahlS.Z-Leprfa/Leprfa rats, Goto–Kakizaki rats, obese spontaneous hypertensive rat (Koletsky rat), and POUND mice [11].

## Development of MetS Model

### Diet-induced models

Diet plays an essential role in developing the clinical manifestations of MetS. Diet can affect metabolism and body regulation through hormones, glucose metabolic pathways, and lipid metabolism [7]. Numerous studies related to metabolic disorders using animal models have been conducted. They used various types of diet and their specific composition, either in the form of mono-diets, such as high-fructose diet, high-sucrose diet, high-fat diet, or the respective combinations of a high-fructose high-fat diet or high-sucrose high-fat diet.

### High-carbohydrate diet models

**Figure-1:**
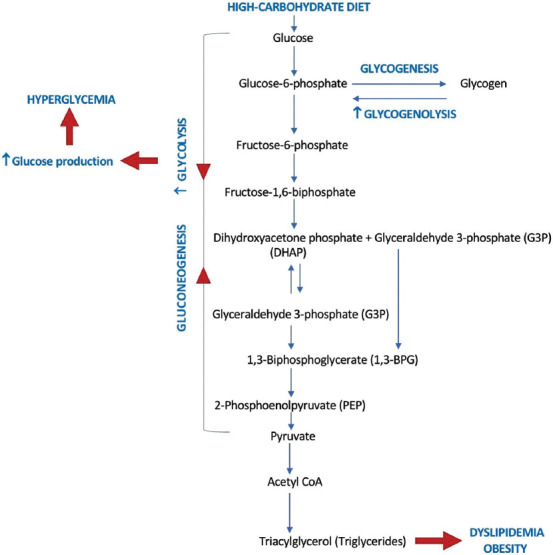
The pathways of glucose metabolism [12].

Carbohydrate is the primary source of energy for the body, which is more readily metabolized than fat. Individuals eating high-carbohydrate diets but with low physical activity tend to store excess energy and develop overweight and obesity. Carbohydrate intake that exceeds energy requirements will increase blood glucose concentrations (Figure-1) [12] and trigger insulin to be secreted from the pancreas and allow cells to uptake glucose. Long-term excessive carbohydrate consumption can cause obesity, which affects insulin resistance.

Recently, Asia’s dietary habits have changed with increased intake of refined carbohydrates, sugars, and saturated fats, while the prevalence of MetS is on the rise, especially in Asia [13]. The intake of refined carbohydrates, such as bread, pasta, and sweeteners in drinks, increases the risk of obesity and type 2 diabetes mellitus. However, a high-carbohydrate high-fiber diet helps to prevent cardiovascular diseases and MetS [6,7,13-15].

The simplest form of carbohydrate molecules contains one sugar molecule called monosaccharides, such as glucose, galactose, and fructose. Two monosaccharides joined together are called disaccharides, such as sucrose, which consist of glucose and fructose [16]. The composition and combination of a high-carbohydrate diet are important factors to be considered in developing MetS models. Basic dietary models applied in experimental conditions are the type of carbohydrates, such as, fructose and sucrose.

### High-fructose diet models

Fructose is commonly known as fruit sugar, which is frequently used as a flavor enhancer in foods. Fructose is an intermediate molecule in glucose metabolism and is rapidly absorbed and metabolized by the liver. Its concentration in peripheral blood is less than that of glucose. Its metabolism differs from glucose in ways that make energy consumption likely to increase [6,17]. A small fructose quantity produces a lower glycemic response to substitute sucrose and starch in the diet of patients with diabetes [7,17,18].

**Figure-2:**
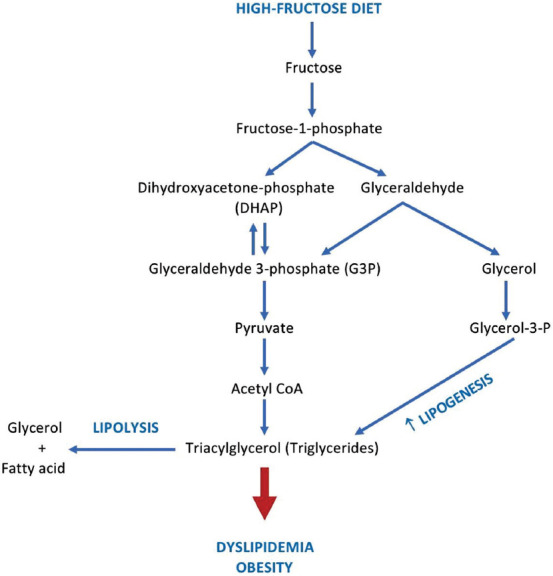
The pathways of fructose metabolism [19].

Nevertheless, fructose intake is large worldwide, which comes from food and beverage sweeteners. Large influx of fructose into the liver causes the accumulation of triglycerides and cholesterol due to the stimulating effects of lipogenesis (Figure-2) [19], which reduces insulin sensitivity leading to insulin resistance and glucose intolerance [17]. A diet con­taining 60% fructose of total calories causes hypertension [20] and increases fasting blood sugar, weight gain, and dyslipidemia [6,21-23]. Several stud­ies have shown that a high-fructose diet can induce MetS symptoms and is an essential factor in the devel­opment of fatty liver (Table-1) [20-28]. The study by Di Luccia *et a*l. [24] has shown that 8 weeks of induction of a high-fructose diet could induce the early symptoms of obesity. The administration of this diet significantly increases body energy, fat, and plasma levels of non-esterified fatty acids (NEFA), a marker of insulin resistance.

**Table-1:** Effects of high-fructose diet on MetS in rat models.

Diet variation	Duration	Strain	Age at induction	Weight (gram)	Components of MetS				Reference

					G	H	O	D	
High-fructose diet (60%)	16 weeks	Male SD rats	-	540-560	√	-	√	√	[21]
High-fructose diet (60%)	6 weeks	Male Wistar rats	-	100-110	√	-	√	√	[22]
High-fructose diet (60%)	6 weeks	Male Wistar rats	-	234	√	-	√	√	[23]
High-fructose diet (60%)	8 weeks	Male SD rats	7 weeks	270	√	√	√	√	[24]
30% fructose in drinking water	8 weeks	Male Wistar rats	-	104-120	√	√	√	√	[25]
20% fructose solution	8 weeks	Male Wistar rats	-	180-200	√	√	√	√	[26]
20% fructose solution in drinking water	15 weeks	Male Wistar rats	3 weeks	280-310	√	-	-	√	[27]
15% fructose in drinking water continued with high-fructose diet (55%)	8 weeks 2 weeks	Male Wistar rats	-	150-250	√	-	√	√	[28]

G=Hyperglycemia, H=Hypertension, O=Obesity, D=Dyslipidemia SD=Sprague Dawley

A high-fructose diet can also be delivered by administering 20-30% fructose in drinking water [25-28]. Nonetheless, these methods show different results. Studies by Ramirez-Higuera *et al*. [25] and Kubacka *et al*. [26] on male Wistar rats for 8 weeks have shown increased fasting blood sugar, increased body weight, hypertension, and dyslipidemia. However, the study by Korkmaz *et al*. has reported the incidence of increased fasting blood sugar and dyslipidemia in male Wistar rats treated with 20% fructose in drinking water for 15 weeks [27].

Chaturvedi *et al*. [28] modified the high-fructose diet by administering a combination of 15% fructose in drinking water for 8 weeks and a high-fructose diet (55% fructose) for 2 weeks. The results showed an increase in fasting blood sugar, body weight, and the incidence of dyslipidemia.

### High-sucrose diet models

Sucrose is a disaccharide consisting of one fructose molecule and one glucose molecule. Similar to fructose, sucrose serves as a food and beverage sweetener. When it is consumed, sucrose is broken down into glucose and fructose by sucrase. The uptake of glucose in glucose metabolism is negatively regulated by phosphofructokinase, causing a continuous inflow of fructose to the glycolytic pathway. Excess fructose will be converted to fat in the liver, and fructose is a better substrate than glucose for fatty acid synthesis and is the main ingredient that contributes to the development of MetS in animals after consuming sucrose [7]. Acosta-Cota *et al*. [29] have conducted a study on male Wistar rats, which received drinking water containing sucrose in various concentrations and duration, namely, 30% sucrose for 12, 16, and 20 weeks and 40% and 50% sucrose for 20 weeks. The results showed a significant increase in the rats’ body weight who received 40% and 50% sucrose. However, these results were not observed in the group of rats that received 30% sucrose (Table-2) [29, 30]. This study showed an increase in blood glucose level, the percentage of intra-abdominal fat, and the incidence of dyslipidemia in all groups. The administration of 30%, 40%, and 50% sucrose reduced the rats’ food intake as the taste of sweet drinks and high-carbohydrate levels affected the central nervous system, which produces dopamine and leptin. It decreases appetite and increases the satiety and hedonic responses to get a higher intake of carbohydrates [29]. The higher the sugar concentration in beverages, the higher the hedonic response, resulting in higher sucrose consumption and less food intake. In addition, high-sucrose supplementation causes hyperglycemia, glucose intolerance, and possibly insulin resistance [30].

**Table-2:** Effects of high-sucrose diet on MetS in rat models.

Diet Variation	Duration	Strain	Age at induction	Weight (gram)	Components of MetS				Reference

					G	H	O	D	
30% sucrose in drinking water 40% and 50% sucrose in drinking water 30% sucrose in drinking water	12, 16, and 20 weeks	Male Wistar	4 weeks	80	√	-	√	√	[29]
	20 weeks				√	-	√	√	
	20 weeks				√	-	√	√	
	16 days	Male Wistar	12 days	496	-	√	-	-	[30]
	7 months			419,67	-	√	-	√	

G=Hyperglycemia, H=Hypertension, O=Obesity, D=Dyslipidemia; SD=Sprague Dawley

Villegas-Romero *et al*. [30] administered 30% sucrose in drinking water to two groups of male Wistar rats with different durations. The administration of 30% sucrose in drinking water in the first group started on the 12^th^ day after birth for 28 days (short-term sucrose group), whereas the second group received the solution up to 7 months (long-term sucrose [LTS] group). The results showed an increase in blood pressure in both groups. Meanwhile, for the LTS group, an increase in visceral fat, hyperinsulinemia, hypertriglyceridemia, and Homeostatic Model Assessment of Insulin Resistance was also observed [30]. This study showed that administering a specific diet from an early age is an essential factor in developing hypertension. The administration of sucrose in a relatively short time since post-birth and the weaning period (short window) can create adult rats having hypertension. The possible underlying mechanisms are as follows: (1) Through the effect of insulin, which may increase endothelin-1 concentrations and inhibit endothelial nitric oxide synthase (eNOS); (2) through an increase in free fatty acids, especially oleic acid, whose levels are related to eNOS activity; and (3) through oxidative stress [30]. Fructose appears to be superior to sucrose in inducing MetS, as fructose is present as a free molecule, whereas sucrose consists of 50% fructose and 50% glucose [7].

### High-fat diet

Fat is one of the three main macronutrients and is the most calorically dense macronutrient [31]. Different types of high-fat diets have been used to develop MetS models, either from animal-derived fats, such as lard or beef tallow, or from plant oils, such as corn oil, soybean oil, and olive or coconut oil. Diet models containing 30-70% fat increase body weight; cause hyperglycemia, insulin resistance, and dyslipidemia; and increase free fatty acids in the blood (Table-3) [32-36].

**Table-3:** Variations of compositions of macronutrients in high-fat diet.

Compositions of macronutrients			Reference

Carbohydrate	Protein	Fat	
18%	12%	70% (8% from soya oil, 62% from lard)	[32]
20%	20%	60% (lard, casein)	[33]
28%	21%	51%	[34]
20%	20%	60%	[35]
56%	14%	30% (lard)	[36]

**Figure-3:**
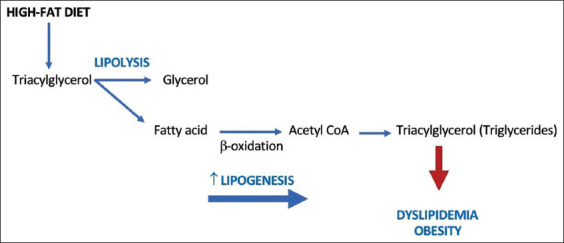
The pathways of lipid metabolism [37].

In lipid metabolism, lipid triglycerides are hydrolyzed into glycerol and three fatty acids, which freely diffuse into the bloodstream. Plasma fatty acids are major substrates for hepatic very low-density lipoprotein (VLDL)-triglyceride production. The administration of a high-fat diet will increase VLDL formation, which helps distribute triglycerides (Figure-3) [37]. A high VLDL cholesterol level leads to obesity, dyslipidemia, and a buildup of cholesterol in arteries (Table-4) [8,32-35,38]. The accumulation of triglycerides in the liver can cause insulin resistance [7]. The administration of a high-fat diet also affects NEFA levels, resulting from the breakdown of triglycerides ingested in the diet. In obesity, circulating levels of NEFAs are elevated, and augmented levels are inversely correlated with insulin sensitivity [36].

**Table-4:** Effects of high-fat diet on MetS in rat models.

Diet variation	Duration	Strain	Age at induction	Weight (gram)	Components of MetS				Reference

					G	H	O	D	
High-fat diet (45%)	17 weeks	Male Wistar	7 weeks	190	√	-	√	-	[8]
		Male SD	7 weeks	208	√	-	√	-	
High-fat diet (45%)	15 weeks	Male Wistar	60 days	-	√	-	√	√	[38]
High-fat diet (70%)	12 weeks	Male SD	6-7 weeks	180-220	√	√	√	√	[32]
High-fat diet (60%)	10 weeks	Male SD	6 weeks	157,5	√	-	√	-	[33]
High-fat diet (51%)	8 weeks	Male SD	90 days	458-494	√	-	√	√	[34]
High-fat diet (60%)	12 weeks	Male SD	3 weeks	-	√	√	√	√	[35]
High-fat diet (30%)	20 weeks	Male Wistar	-	409	√	√	√	√	[8]

G=Hyperglycemia, H=Hypertension, O=Obesity, D=Dyslipidemia; SD=Sprague Dawley

### Combination of High-Fat and High-Carbohydrate Diet Models

A high-fat, high-carbohydrate diet is a diet model that most closely resembles the current trend toward changing eating habits. Increasing diets that contain large amounts of saturated fat and refined carbohydrates are an essential factor in triggering MetS, obesity, and type 2 diabetes mellitus. Fructose and sucrose, widely used as food or beverage sweeteners, provide less satiety than other sugar types, thus encouraging excessive intake [39].

This combination diet provides several advantages. The increase in triglycerides mainly occurs due to high-fructose diets, whereas obesity occurs due to high-fat diets. This combination diet was associated with an increase in plasma cholesterol levels, a decrease in HDL cholesterol, and a 2-fold increase in liver weight in rats (Table-5) [39-45]. A study has shown the accumulation of lipids in the myocardium, left ventricular hypertrophy, and morphological liver damage. One of the advantages of using a high-fat, high-carbohydrate diet is that damage to heart tissue occurs relatively quickly; thus, examining the morphological, biochemical, and functional properties of cardiovascular pathogenesis in detail is possible [6].

**Table-5:** Effects of high-fat high-carbohydrate diet on MetS in rat models.

Diet variation	Duration	Strain	Age at induction	Weight (gram)	Components of MetS				Reference

					G	H	O	D	
High-fat diet (22,5%)+25% glucose in drinking water	20 weeks	Male Wistar	8 weeks	280-310	√	-	√	√	[39]
High-fat diet (22,5%)+25% fructose in drinking water					√	-	-	√	
High-fat diet, high-carbohydrate diet	14 weeks	Male Wistar	8 weeks	130	√	√	√	√	[40]
High-fat diet, high-carbohydrate diet+15% fructose in drinking water	16 weeks	Male SD	-	220-250	√	√	-	√	[41]
High-fat diet (66,28%)+60% fructose in drinking water	4 weeks	Male Wistar	-	-	√	-	-	√	[42]
High-fat diet (21,4%)+25% fructose in drinking water]	8 weeks	Male Wistar	-	185-200	√	-	-	-	[43]
High-fat diet+25% fructose in drinking water	16 weeks	Male Wistar	3 months	200-250	√	√	-	√	[44]
High-fat (35%), high-carbohydrate (45%) diet	16 weeks	Male Wistar	8 weeks	180-220	√	√	√	√	[45]

G=Fasting hyperglycemia, H=Hypertension, O=Obesity, D=Dyslipidemia SD=Sprague Dawley

Excessive calorie intake induces fat accumulation leading to deregulation of adipocyte function, resulting in inflammation and free radical production. The inflammatory process plays a key role in insulin resistance, obesity, and glucose intolerance [39]. Moreno-Fernandez *et al*. [39] combined a high-fat diet with a high-carbohydrate one, using additional 25% glucose and 25% fructose in drinking water, respectively, in two groups. The results showed increased body weight and abdominal circumference in the glucose group, but not in the fructose group. From most studies using a high-fructose diet, the amount that could induce weight gain is more than 30% fructose [19,22,24].

### High Sodium Chloride (NaCl) and High-Fructose Diet

A high NaCl (salt) diet is used to induce hypertension in rats, but it can also be used to develop MetS models. The administration of high NaCl content (8% NaCl) for 2 weeks increased systolic blood pressure, plasma glucose levels, and liver gluconeogenesis [6]. Recent studies have suggested that a high-salt diet activates processes that result in fructose generation in the liver (endogenous fructose production) [46].

**Figure-4:**
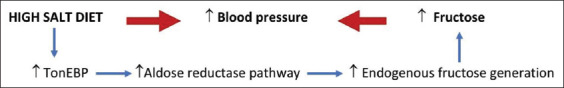
Interaction between high salt diet and endogenous fructose generation [46].

The combination of a high NaCl diet with a high-fructose one (60%) increased blood pressure and signs of renal damage [6]. Strong interactions exist between salt and fructose intake. Salt intake may increase thirst, which might encourage the intake of sugary beverages, thereby enhancing fructose intake. High-salt diets are associated with a higher risk of developing diabetes mellitus, regardless of calorie intake. However, the association of salt with fructose may be more complex. Fructose enhances salt absorption in the gut and kidneys and enhances intracellular angiotensin formation. A high-salt diet will activate tonicity-responsive enhancer binding protein (TonEBP), a transcription factor that promotes kidney inflammation and osmolyte production in response to inflammation and osmotic stress. In turn, TonEBP activates the aldose reductase pathway, leading to endogenous fructose generation; this indicates a complex interplay in which salt and fructose synergize together to raise blood pressure (Figure-4) [46].

### Chemical-Induced Models

MetS models can be developed using drugs or chemical substances, such as glucocorticoids, antipsychotics, alloxan, and streptozotocin (STZ) [6,7]. This method is suitable for drug-related MetS studies, but it needs a longer induction duration to meet the criteria for MetS models [46].

### Glucocorticoids Induction

Glucocorticoids are essential steroid hormones secreted from the adrenal gland in response to stress and have been widely prescribed to treat inflammatory disorders and autoimmune diseases. Unfortunately, the therapeutic benefits of glucocorticoids are limited due to side effects associated with high-dose and long-term usage. These side effects include osteoporosis, skin atrophy, diabetes, abdominal obesity, glaucoma, cataracts, avascular necrosis and infection, growth retardation, and hypertension [47].

**Figure-5:**
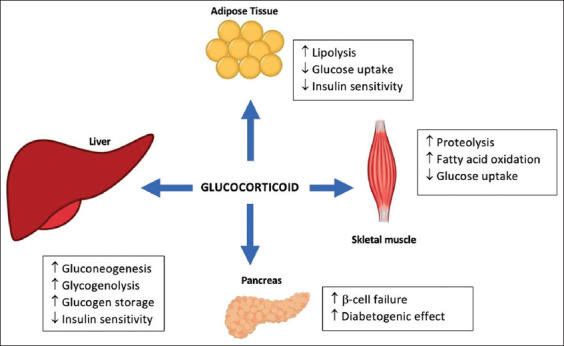
Effects of glucocorticoid on body metabolism [48].

Glucocorticoids contribute to glucose metabolism in the liver, skeletal muscle, adipose tissue, and the pancreas (Figure-5) [48]. Glucocorticoids regulate the expression of major gluconeogenic enzymes in the liver, such as phosphoenolpyruvate carboxykinase, glucose-6-phosphatase, and tyrosine aminotransferase. In skeletal muscles, glucocorticoid excess can inhibit the translocation of glucose transporter type 4 to the plasma membrane in response to insulin, resulting in insulin resistance. Glucocorticoids induce adipocyte differentiation, leading to increased adiposity and insulin resistance [49]. In the pancreas, glucocorticoids induce hyperglycemia by inhibiting glucose-stimulated insulin secretion from b-cells [50]. Glucocorticoid induction can be administered to animal models by oral feeding, intraperitoneal injections, or surgical implantation of glucocorticoid pellets. These methods will result in almost similar MetS components: Weight gain, abdominal fat accumulation, severe fasting hyperglycemia, insulin resistance, impaired glucose tolerance, hypertension, and dyslipidemia [7].

### Antipsychotics Induction

Antipsychotics form the mainstay of treatment for patients with schizophrenia, bipolar disorder, and other mental disorders. Despite the benefits of these drugs, some animal studies have shown that especially second-generation antipsychotics (SGAs) are associated with weight gain, lipid abnormalities, increased visceral fat, impaired glucose tolerance, and insulin resistance. Thereby, they contribute to the development of MetS and insulin resistance [51]. Among them, olanzapine and clozapine appear to have the highest tendency to disturb glucose metabolism compared with other antipsychotics [52]. The metabolic disturbance mechanisms by SGAs involve both peripheral and central mechanisms. Weight gain is a common side effect of SGAs. The inhibition of hypothalamic 5HT2C and H1 receptors will result in increased appetite stimulation [53]. Liu *et al*. [53] have compared olanzapine (3 mg/kg, twice a day) with clozapine (20 mg/kg, twice a day) for 9 weeks to observe the metabolic profile of rats. The study has shown that olanzapine caused weight gain and elevated hepatic lipid levels, which could induce insulin resistance. In addition, clozapine caused weight gain and had an influential direct role in lipid accumulation and insulin secretion deficiency, impairing glucose tolerance.

### Alloxan and STZ Induction

Alloxan (5,5-dihydroxyl pyrimidine-2,4,6-trione) is a toxic glucose analog derived from uric acid oxidation. It can induce diabetes in rodents by producing reactive oxygen species (ROS) that cause the destruction of b-cells in the pancreas. Its 5-carbonyl group is reactive to thiol groups, indicating a structure-function relationship in alloxan toxicity. Its hydrophilic structure is similar to that of glucose, enabling alloxan to be transported into the pancreas involving glucose transporter 2 (GLUT2). In b-cells of the pancreas, alloxan inhibits glucokinase, the most sensitive thiol enzyme, which has an essential role as a glucose sensor in the pancreas and liver [54,55].

STZ (2-deoxy-2-(3-(methyl-3-nitrosoureido)-D-glucopyranose) is a fungal antitumor antibiotic derived from *Streptomyces achromogenes*. STZ has properties similar to those of alloxan that contributes to inducing diabetes in animal models, but it has a more selective toxic effect on b-cells of the pancreas. The most important mechanism of STZ in inducing diabetes is DNA alkylation. In addition, STZ may damage the b-cell membrane and break DNA strands, leading to cell death. STZ also generates ROS resulting in diabetogenic effects [54].

### Combination of High-Fat Diet and STZ Induction

The administration of a high-fat diet along with STZ induction can develop a type 2 diabetes mellitus model that mimics diabetes mellitus in humans. A high-fat diet will induce insulin resistance in peripheral tissues related to lipotoxicity, while low-dose STZ (25-30 mg/kg) will induce a mild defect in insulin secretion. The combination of induction can produce metabolic changes that mimic type 2 diabetes mellitus in humans [56,57]. In the study by Jakovljevic *et al*. [56] male Wistar rats received a high-fat diet and 25 mg/kg STZ. These combinations can develop MetS components in rats, which had an increase in body weight, hyperglycemia, and hypertension.

Furthermore, in the study by Rohman *et al.*, [57] Sprague-Dawley male rats received a high-fat high-sucrose diet and 30 mg/kg STZ to maintain a hyperglycemic state. This experiment produced an animal model representing the complete components of MetS (Table-6) [56,57]. The administration of a high-fat diet induces insulin resistance by activating protein kinase C induced by a high level of free fatty acid that interfered with the activation of insulin receptor substrate. Dyslipidemia occurs due to an increase in free fatty acids, resulting from the combination diet. Free fatty acids induce inflammation and increase the metabolism of triglycerides and cholesterol. Hypertension occurs due to increased peripheral resistance and the activation of the renin-angiotensin-aldosterone system [57].

**Table-6:** Effects of high-fat diet/high-fat high-sucrose diet with streptozotocin on MetS in rat models.

Diet variation	Duration	Strain	Age at induction	Weight (gram)	Components of MetS				Reference

					G	H	O	D	
High-fat diet (25%)+STZ (25 mg/kg)	4 weeks	Male Wistar	6 weeks	200	√	√	√	-	[56]
High-fat diet (40%), high-sucrose diet (20%)+STZ (30 mg/kg)	8 weeks	Male SD	8-12 weeks	230-340	√	√	√	√	[57]

G=Hyperglycemia, H=Hypertension, O=Obesity, D=Dyslipidemia; SD=Sprague Dawley

## Conclusion

Developing an ideal model of MetS is challenging. Selecting animal models that are appropriate to represent the clinical manifestations of human conditions are essential. Several factors must be considered in developing rat MetS models, including strain, gender, age, induction type, and induction duration. In addition, the models should be reproducible, reliable, and affordable. The development of proper models will contribute to the successful application of research findings to the clinical setting.

## Authors’ Contributions

SG: Conception of the specific review, collected literature, and wrote the original manuscript. AA and VS: Contributed to the review, editing, and supported in the supervision. All authors read and approved the final manuscript.
